# Recent advances in biopolymer-based antibacterial food packaging with natural antimicrobial agents

**DOI:** 10.1039/d6ra08017k

**Published:** 2026-10-21

**Authors:** Ertao Du, Yushuo Yang, Yuewei Xi, Yunxuan Weng

**Affiliations:** a College of Advanced Materials and Future Technology, Beijing Technology and Business University Beijing 100048 China xiyuewei@btbu.edu.cn wyxuan@th.btbu.edu.cn

## Abstract

The growing emphasis on environmental protection, together with plastic-restriction policies and dual-carbon goals, has driven the food packaging industry to replace non-degradable petrochemical polymers with renewable bio-based materials such as cellulose, starch, and chitosan. Incorporating natural antimicrobials into biodegradable polymer matrices offers a promising strategy to develop active packaging that inhibits microbial growth, ensures food safety, and extends shelf life. This review systematically summarizes recent advances in antibacterial food packaging based on biopolymers incorporated with natural antimicrobial agents, which are classified as plant-derived (spices, herbs, and essential oils), animal-derived (chitosan, lysozyme, lactoferrin, and lactoperoxidase), and microbial-derived (bacteriocins, nisin, bacteriophages, reuterin, and pediocin) agents. Their antimicrobial mechanisms and packaging applications are elaborated, alongside four typical antibacterial packaging strategies. Notably, chitosan, starch and sodium alginate are promising matrices for chemical modification; cross-linking, grafting and esterification can improve matrix-agent interfacial compatibility to tailor active-compound release kinetics. Finally, the remaining challenges, including matrix-agent compatibility, migration of active agents, and safety concerns, as well as future directions toward industrial-scale application, are highlighted. This review provides a comprehensive reference for developing natural antimicrobial food packaging to reduce environmental pollution while improving food quality and safety.

## Abstract 2

Schematic illustration of biopolymer-based antibacterial food packaging with natural antimicrobial agents.

## Graphical abstract

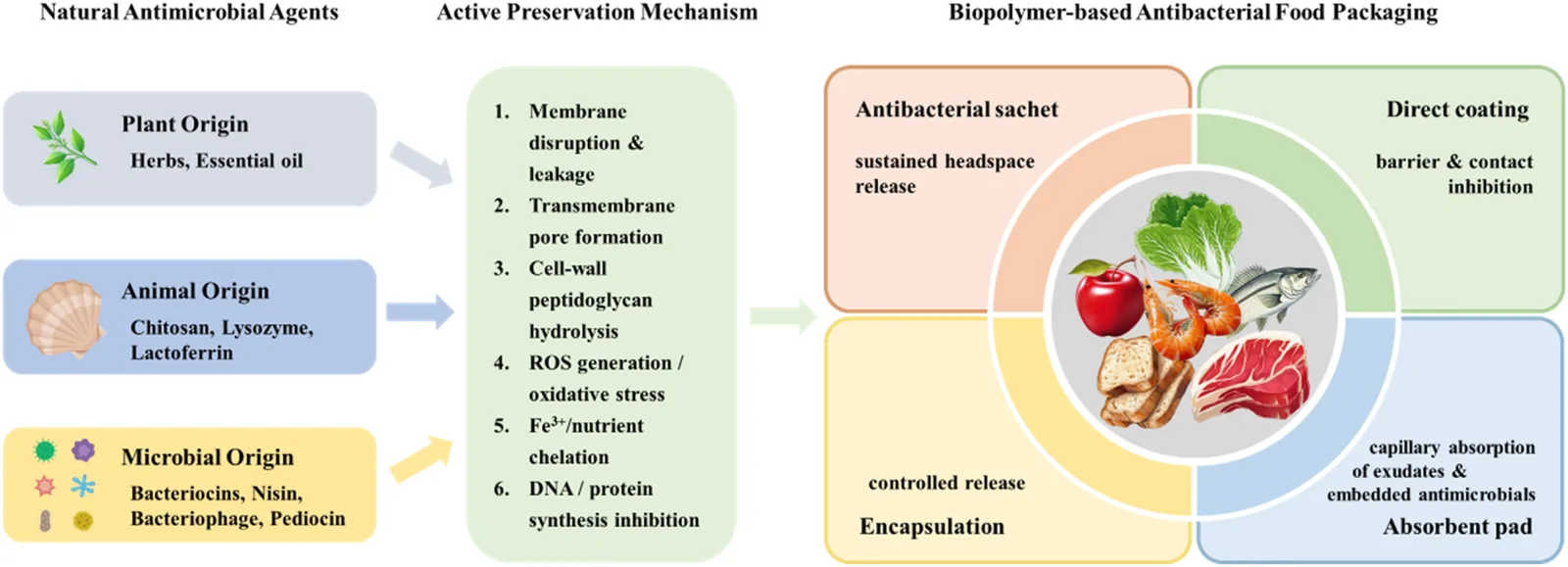

## 1 Introduction

Food packaging materials refer to plastic, fiber, glass and other materials used for packaging and holding food. Food packaging can protect the quality of food during storage and transportation from the impact of the external environment, and provide consumers with information about the composition of the product, so that consumers can better understand the product and compare it with their own needs. The complete goals of food packaging are: (1) to inhibit microbial growth, (2) to maintain its stability against environmental hazards and to resist oxidation, (3) to mask unpleasant odors while maintaining flavor, (4) to continuously deliver nutrients, (5) to help filter and accumulate elements, and (6) to act as sensor carriers.^1^ In recent years, environmental pollution has increasing attracted attention from countries around the world. With the increasing emphasis on environmental protection by the country, new policies such as “plastic restriction orders” and “dual carbon goals” have been introduced, which have also put forward higher requirements for various industries. For the food industry, the choice of packaging materials has an indispensable impact on the environment. For consumers, the natural, nutritious and healthy food is also very important, which requires that the food is not polluted by the external environment during storage and transportation, and the freshness of the food itself needs to be guaranteed. Under the conditions of the environment and consumer preference trends, the selection of packaging materials becomes extremely important.

Most of the traditional food packaging materials are based on petrochemical polymers, although they have the advantages of lightweight quality and good mechanical properties, but most of these polymers are non-degradable, which is one of the main sources of “white pollution”. Moreover, some plasticizers can also be combined with polymers to improve the performance of packaging, but these substances have certain side effects on human health. Under various factors, researchers have begun to search for biodegradable, recyclable natural bio-based polymers that can replace petrochemical based packaging materials. Bio-based polymer packaging material is a new type of packaging material prepared from natural and renewable polymers such as cellulose, starch and chitosan. This kind of packaging materials can alleviate the pressure of white pollution on the environment and solve the problem of resource shortage to a certain extent. However, the mechanical properties of these packaging itself, such as tensile strength, breaking strength, and some other physical and chemical properties such as oxygen barrier, are not enough to allow it to be directly applied to food packaging, so it is necessary to develop certain technologies to modify them.

Active packaging is one of the research hotspots in new food packaging. Active packaging systems mainly include active release system (release of antioxidant and antibacterial substances), active absorption system (oxygen absorption, moisture absorption, CO_2_ absorption) and other systems.^2^ Biobased active packaging materials are based on biodegradable materials, and add active substances with different compositions and functions to the substrate to prepare food packaging with different functions, thereby ensure food quality and safety. According to the consumer's pursuit of food quality, it is particularly important to add antibacterial substances to the bio-based packaging matrix, develop a food packaging material with antibacterial function, inhibit the growth and reproduction of microorganisms, and protect the internal food. The use of antibacterial packaging reduces the growth of microorganisms inside the packaging, improves food quality and safety, ensures a longer shelf life. It is by far an effective way to control the proliferation of bacteria and fungi that cause food spoilage.

Numerous existing reviews have summarized the performance, preparation processes, and food-preservation efficacy of biopolymer-based antibacterial food packaging. However, most previous reviews either focus only on a single category of antimicrobial agents or merely describe the fabrication and application phenomena of composite films. Critical discussions regarding matrix-antimicrobial interfacial compatibility, chemical-modification strategies for biopolymer matrices, and the correlations between chemical-modification approaches and the release kinetics of active substances remain scarce. Meanwhile, few reviews systematically compare four practically implementable antibacterial-packaging modes (antibacterial sachets, direct coating, encapsulation, and absorbent-pad systems), nor do they establish links between material-modification strategies and real-world industrial bottlenecks such as active-agent-migration risks and food-matrix compatibility. To fill these research gaps, the present review categorizes natural antimicrobial agents by their biological origins and elaborates their antibacterial mechanisms. Fig. 1 shows the classification of antimicrobial agents for active food packaging applications. It further identifies biopolymer matrices with high modification potential, including chitosan, starch, and sodium alginate, and illustrates how chemical strategies such as cross-linking, grafting, and esterification improve interfacial compatibility and enable the regulation of release behaviours. Four representative technical routes for antibacterial packaging are systematically summarized, and prevailing practical obstacles as well as prospects for industrial-scale implementation are analysed. Accordingly, this review delivers a differentiated integrated perspective and provides more targeted references for the rational design and subsequent industrial translation of biopolymer-based antibacterial active food-packaging systems.

**Fig. 1:**
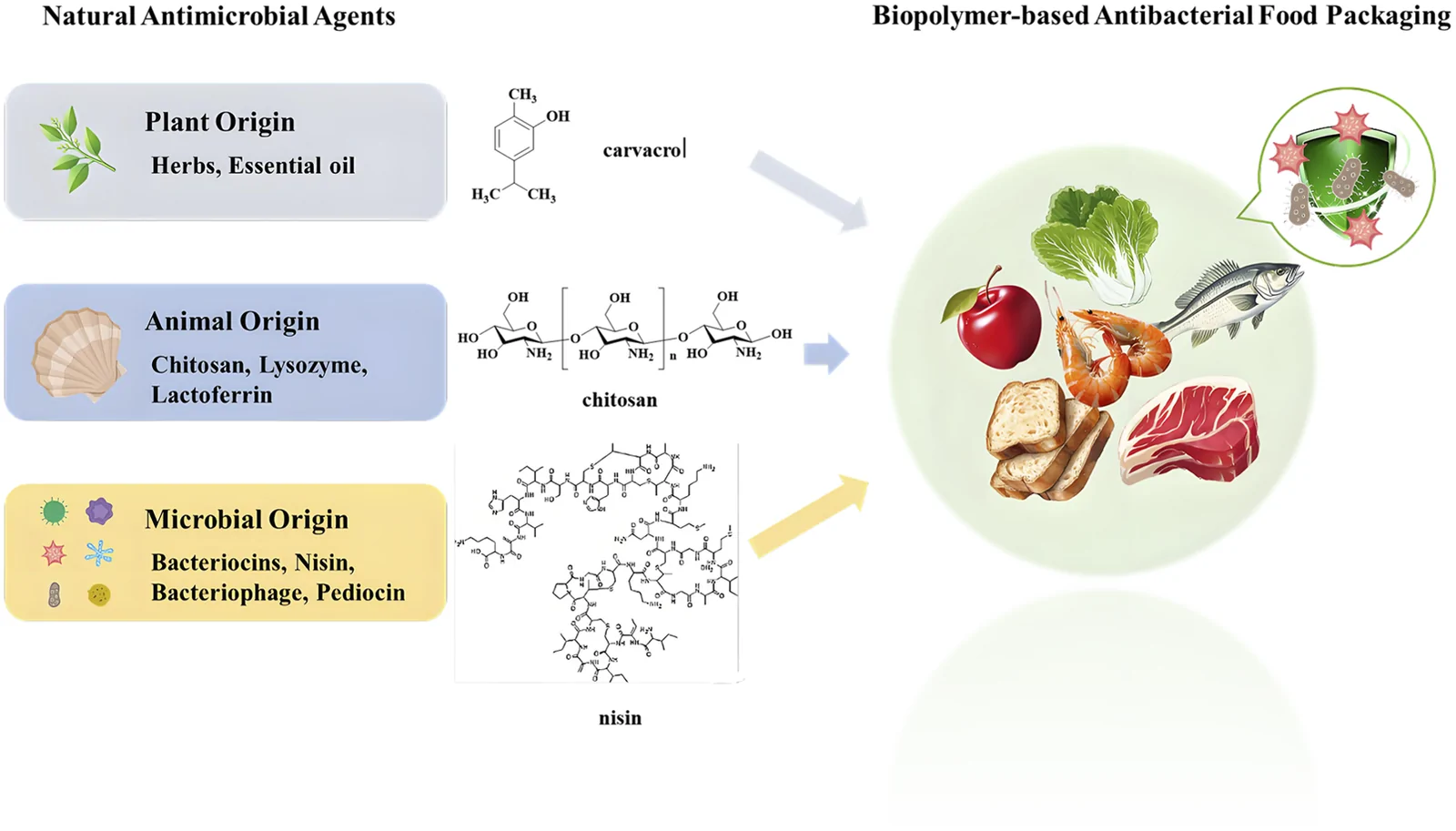
Classification of antimicrobial agents for active food packaging applications.

## 2 Types of antibacterial agents

The proliferation of bacteria, fungi and food-borne pathogens in food can seriously affect the color and texture of food. In addition, the proliferation of some bacteria can also lead to various diseases, posing a serious threat to the health of consumers. Adding antibacterial agents to active packaging can effectively inhibit the growth of microorganisms, reduce the deterioration of food, extend the shelf life of food, and ensure food safety. There are also many types of antibacterial agents that can be incorporated into active packaging materials. At present, researchers are committed to seeking natural antibacterial agents to reduce the change of antibacterial agents on food flavor and quality, and to minimize the impact on human health while improving antibacterial efficacy. Different natural antibacterial agents have different structures and resulting in different antimicrobial mechanisms. For example, polyphenols contain hydroxyl groups in their structure, which can interact with the plasma membrane of bacteria to destroy the permeability of the cell membrane, leading to the leakage of intracellular components, the outflow of nutrients, and the loss of cell activity, thus achieving the purpose of bacteriostasis.^3^ In this mechanism, the hydroxyl group may act as a proton exchanger, resulting in a decrease in pH gradient across the plasma membrane and cell death.^4^ According to the different sources of natural antibacterial agents, they can be divided into the following three categories: plant sources, animal sources and microbial sources.

### 2.1 Antibacterial agents of plant origin

#### 2.1.1 Spices and herbs

In past studies, spices and herbs and their extracts have been shown to have antibacterial properties. Most compounds are found in different parts of plants, namely roots, bark, and flowers, and are mainly used in complete, crushed, or encapsulated forms in food packaging.^5,6^ In addition to their significant antibacterial capacity, Pisoschi *et al.* have also found that they have a strong antioxidant capacity that helps to inhibit lipid oxidation,^7^ which is attributed to the presence of secondary metabolites such as phenolic acids, saponins, tannins, alkaloids, alkenyl phenols, glycoalkaloids, flavonoids, sesquiterpenes, and terpenoids.^3,8^ Among them, thymol (thyme), eugenol (clove), cinnamaldehyde (cinnamon) and carvacrol represent signature bioactive compounds responsible for their functional performance. Phenolic hydroxyl (–OH), aldehyde (–CHO) and alkene groups within these molecules serve as key functional moieties mediating bio-activity. For microbial membranes, hydrophobic aromatic skeletons enable insertion into the lipid bilayer, while polar functional groups trigger membrane depolarization, increase membrane fluidity and permeability, provoke leakage of intracellular electrolytes, metabolites and cytoplasmic materials, and eventually induce microbial cell death.^3^ Aldehyde groups can additionally react with amino-sulfhydryl sites of membrane-bound proteins, denature protein conformation and further impair membrane-associated physiological processes.

These plant extracts reduce the risk of foodborne illness for consumers by inhibiting microbial action.^9^ Although their safety can cause consumer concern, it is said that antibacterial agents extracted from most plants are considered GRAS (Generally Recognized as Safe). (United States FDA evaluation of food additives safety indicators: GRAS certification: english abbreviation for Generally Recognized as Safe).^10^ The research results of Jose Maria *et al.* showed that five spices and herbs (thyme, rosemary, cloves, oregano and basil) had inhibitory effects on six microorganisms, including *Listeria monocytogenes*, *Bacillus cereus*, fungi and yeast. Moreover, the cloves had the most obvious inhibitory effect.^11^ In addition, Ferriccioni *et al.* found that dried thyme can reduce the growth and proliferation of aerobic thermophilic microorganisms, molds and yeasts by more than 90%.^12^ In particular, the inhibitory effect on microorganisms in meat is more prominent. These examples all demonstrate the powerful antibacterial and antioxidant properties of spices and herbs in food.^13^ Therefore, spices and herbs have the potential to completely replace the chemical additives that currently exist, so their application in antibacterial food packaging should be vigorously promoted.

#### 2.1.2 Essential oil

Essential oils, such as oregano essential oil, cinnamon essential oil, clove essential oil, rosemary, lemongrass, *etc.*, have significant inhibitory effects on microbial activity. Essential oil itself is extracted from different parts of plants including leaves, bark, flowers and roots through different processes, and is a volatile secondary metabolite.^4^ Essential oil extraction processes mainly include steam distillation, organic solvent extraction, steam distillation, supercritical fluid extraction and cold pressing.^14^ Compared with chemical or synthetic additives, essential oils contain naturally active compounds, which are added to active packaging as antibacterial agents to suppress microbial activity while also reducing food safety risks.^15,16^

Essential oils are a complex natural mixture consisting of 20–60 components in varying concentrations.^17^ The antibacterial mechanism of essential oils is not uniform; differences in their composition and concentration produce distinct modes of antimicrobial action. At present, the majority of researchers agree that the microbial cell membrane is the primary target of essential oils.^18^ From a chemical perspective, the dominant lipophilic constituents of essential oils—including monoterpenes, sesquiterpenes and phenolic derivatives (*e.g.*, thymol, carvacrol, eugenol and cinnamaldehyde)—possess hydrophobic aliphatic isoprenoid chains or aromatic ring skeletons. These structures drive spontaneous insertion into the hydrophobic acyl-tail region of the bacterial phospholipid bilayer, disrupting the tightly ordered packing of fatty-acid chains. This structural disturbance increases membrane fluidity and induces transient or permanent membrane pores, thereby elevating membrane permeability in a concentration-dependent manner.^19^Fig. 2 shows the antibacterial mechanism of essential oils. Elevated membrane permeability leads to leakage of intracellular electrolytes, protons and low-molecular-weight metabolites, which dissipates the transmembrane pH gradient and proton motive force and thereby inhibits ATP synthesis and cellular energy production. Meanwhile, the inserted hydrophobic components induce conformational changes and denaturation of integral membrane proteins, impairing membrane-associated physiological functions.

**Fig. 2:**
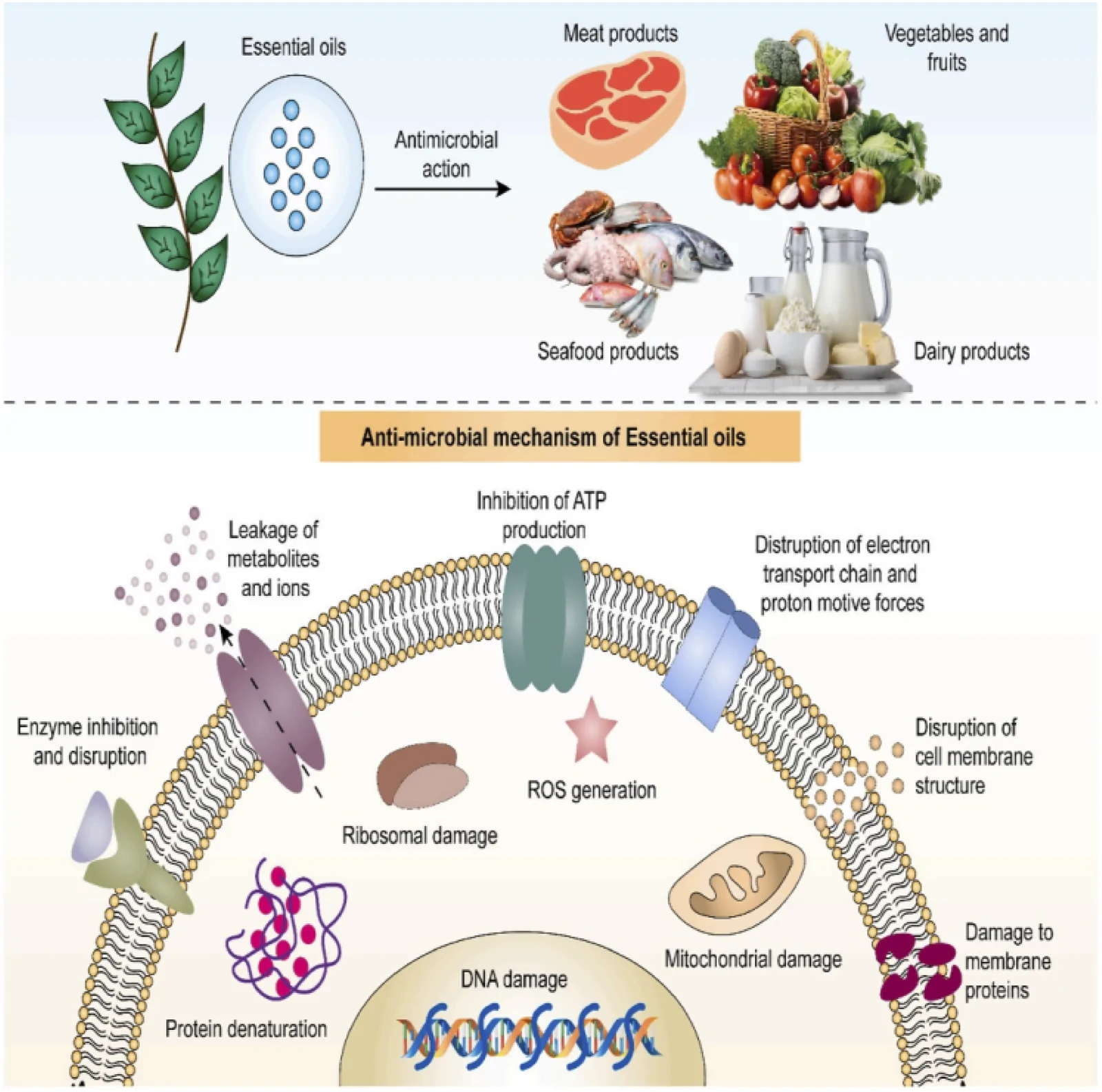
Antimicrobial mechanism of essential oils.^20^ Reproduced from ref. 20 with permission from Elsevier, Rout *et al.*, Recent trends in the application of essential oils: the next generation of food preservation and food packaging, *Trends in Food Science & Technology*, 2022, **129**, 421–439, https://doi.org/10.1016/j.tifs.2022.10.012, copyright 2022 Elsevier Ltd.

Once internalized into the cytoplasm, lipophilic essential-oil constituents target the mitochondrial inner membrane, disrupt the electron transport chain and cause electron leakage, which drives the generation of reactive oxygen species (ROS). Excessive ROS triggers severe oxidative stress including lipid peroxidation, protein denaturation and oxidative DNA damage, ultimately resulting in irreversible microbial cell death.^20^

Currently, there have been many studies on the antibacterial properties of essential oils. For example, Zhang *et al.* found that essential oils extracted from licorice and rosemary can inhibit the activity of *Listeria monocytogenes*, *Escherichia coli*, *Pseudomonas fluorescens* and fluorescens *Lactobacillus* sake.^21^ Linalool, a monoterpene compound isolated from lavender essential oil, has been reported to have strong antibacterial activity against drug-resistant *Klebsiella pneumoniae*.^22^ Garlic essential oil has strong inhibition against *Escherichia coli*, *Staphylococcus aureus* and *Salmonella typhimurium*.^23^ Extracts from several other plants, such as citrus peels, rosemary plants, and lemongrass, are also highly resistant to microorganisms that cause food spoilage.^24,25^

Similarly, essential oils also have significant antibacterial effects on some meat and dairy products. Manuel *et al.* showed that combining thyme essential oil with sodium citrate had good inhibitory properties on microbial activity of whey cheese stored at 5 °C for 21 days, and did not affect the existence of *Lactobacillus* in cheese.^26^ The research results of Karabagias *et al.* showed that essential oil extracts from thyme, cinnamon, laurel and clove showed significant inhibitory effect on monocyte *Listeria monocytogenes* in cheese.^27^ Sobhy *et al.* combined cinnamon essential oil and mustard essential oil with a ratio of 100 ∶ 8 to explore the antibacterial effect of the combination. Such a ratio not only covered up the pungent taste of mustard, but also retained its own antibacterial properties. The results indicated that the combination has a good inhibitory effect on microbial activity.^28^ When the combination of rosemary essential oil and thyme essential oil was applied to beef sausage, the number of microorganisms in it was significantly reduced.^29^ These literature all indicated that whether it is dairy products or meat products, even under low temperature conditions, essential oils show strong antibacterial properties against a large number of bacterial strains. Table 1 provides a merged summary of natural antimicrobial agents (essential oils, plant extracts, animal-derived, and microbial-derived) used in food packaging.

**Table 1:** The merged summary of natural antimicrobial agents used in food packaging^a^

Food	Antimicrobial agent/extract	Biopolymer matrix	Target microorganism	Required EO concentration (%)	Antibacterial rate (%)	Overall rating	References
Meat	Pomegranate peel extract	PEO/chitosan	*Escherichia coli*	1.0–3.0	>90	★★★	30
Chilled beef	Beetroot extract	Pectin (watermelon peel)	Antioxidant/indicator	—	—	★★	31
Strawberry	Apple polyphenols	Sodium alginate	*E. coli*, *S. aureus*	0.5–2.0	85–95	★★★	32
—	Eugenol	Chitosan–pectin–starch	*S. aureus*, *E. coli*	1.0–3.0	>90	★★★	33
Chicken	Thyme essential oil	Gelatin nanofibers	*Campylobacter jejuni*	0.5–2.0	>99	★★★	34
Fish	Clove essential oil	Gelatin–chitosan	*S. aureus*	1.0–2.5	>90	★★★	35
—	*Eucalyptus globulus* oil	Zein	*S. aureus*, *L. monocytogenes*	0.5–2.0	80–95	★★★	36
Sliced sausages	*Eucalyptus globulus* oil	Chitosan	*S. aureus*, *E. coli*, *B. cereus*, *S. enteritidis*	1.0–3.0	>90	★★★	37
Whole beef muscles	Oregano essential oil	Milk protein	*S. aureus*	0.5–2.0	85–95	★★★	38
Pork meat	Oregano essential oil	Starch–chitosan	Total aerobic, coliform	1.0–3.0	>90	★★★	39
Lamb meat	Rosemary essential oil	Cellulose nanofiber/whey protein	Psychrotrophic/total coliform	0.5–2.0	80–90	★★	40
—	Cinnamon essential oil	Starch–carboxymethyl cellulose	*S. aureus*	1.0–3.0	>95	★★★	41
Strawberry	Cinnamon essential oil	Gelatin–starch	*P. commune*, *E. amstelodami*	0.5–2.0	85–95	★★★	42
Fresh pistachio	Cinnamon essential oil	Sago starch	*E. coli*, *S. typhimurium*, *S. aureus*	1.0–3.0	>90	★★★	43
—	Clove essential oil	PLA–PBAT	*E. coli*, *S. aureus*	1.0–3.0	>90	★★★	44
Precooked pork chops	Winter savory EO	Alginate	*L. monocytogenes*	0.5–2.0	80–95	★★★	45
Maize	Black pepper EO	Alginate	*Aspergillus flavus*	1.0–3.0	75–90	★★	46
Guava fruit	Peppermint essential oil	Gelatin–starch	*Alternaria alternata*	0.5–2.0	80–95	★★★	47
Beef loins	Cumin essential oil	Chitosan	*L. monocytogenes*, *E. coli*, *S. typhimurium*	1.0–3.0	>90	★★★	48
Pork chops	Lemon essential oil	Chitosan–collagen	*L. monocytogenes*	0.5–2.0	85–95	★★★	49
Cherry tomatoes	Lavender essential oil	Gelatin	*S. aureus*, *E. coli*, *L. monocytogenes*	0.5–2.0	80–90	★★★	50
Meat	Casein phosphopeptides	Gelatin edible film	Various pathogens	—	—	★★	51
Meat	Ginger oil	Chitosan/montmorillonite	Poultry meat spoilage bacteria	0.5–2.0	80–90	★★	52
Meat	*Mentha spicata* oil	Chitosan coating	Camel meat spoilage bacteria	0.5–2.0	80–95	★★	53
Meat	Rosemary essential oil	Chitosan/montmorillonite	Poultry meat spoilage bacteria	0.5–2.0	85–95	★★★	54
Meat	Thyme essential oil	Gelatin nanofibers	*Campylobacter jejuni*	0.5–2.0	>99	★★★	34
Meat	Grape seed extract	Chitosan coating	Rainbow trout spoilage bacteria	—	—	★★	55
Meat	*Eucalyptus* oil	Chitosan film	Sausage spoilage bacteria	1.0–3.0	>90	★★★	37
Meat	Protocatechuic acid	Gelatin film	Beef spoilage bacteria	—	—	★★	56
Fruits	*Mentha spicata*	CMC–chitosan coating	Strawberry spoilage bacteria	0.5–2.0	80–90	★★	57
Fruits	Cinnamon oil	Encapsulated film	Fruit surface bacteria	0.5–2.0	85–95	★★★	58
Fruits	Limonene	Chitosan–limonene coating	Cucumber postharvest pathogens	0.5–1.5	75–90	★★	59
Fruits	Thyme essential oil	Chitosan coating	Salmon/seaweed burger pathogens	0.5–2.0	80–95	★★★	60
Fruits	Apple polyphenols/AgNPs	Sodium alginate film	Strawberry spoilage bacteria	—	—	★★	32
Dairy and cheese	Garlic essential oil	Dairy packaging	Cheese spoilage bacteria	0.5–2.0	80–90	★★	61
Dairy and cheese	Pomegranate peel extract	Dairy film	Cheese spoilage bacteria	1.0–3.0	>90	★★★	62
Dairy and cheese	Cinnamon oil	Dairy packaging film	Cheese spoilage fungi	0.5–2.0	85–95	★★★	63
Dairy and cheese	Clove leaf oil	Vegetable packaging	Vegetable spoilage bacteria	0.5–2.0	80–90	★★	64
Dairy and cheese	Ginger oil	Vegetable coating	Vegetable spoilage bacteria	0.5–2.0	80–90	★★	65
Dairy and cheese	Blood orange peel pectin	Pectin-based film	Vegetable pathogens	—	—	★★	66
Vegetables	Nisin	Vegetable packaging	Gram-positive bacteria	0.1–0.5	>99	★★★	67

a Overall rating criteria: ★★★ (high) = broad-spectrum inhibition covering ≥3 microbial targets or antibacterial rate >90%; ★★ (medium) = moderate inhibition covering 2 microbial targets or antibacterial rate 80–90%; ★ (low) = narrow-spectrum or antibacterial rate <80%, or efficacy data not reported. Required essential-oil concentration is given as the typical loading range reported in the cited studies; “—” indicates that the original reference did not report a quantitative value.

Compared to chemical additives, essential oils have lower cost, minimal harm to the human body, and excellent antibacterial effect. Therefore, researchers should increase their research on the antibacterial properties of essential oils and their application in food packaging.^17^

### 2.2 Antibacterial agents of animal origin

Proteins and enzymes are the most common natural antibacterial compounds found in animals. These natural compounds include chitosan, lactoperoxidase, lactoferrin, and lysozyme. They have strong antibacterial ability and have great potential in antibacterial food packaging.^17^ Their antibacterial mechanism is similar to that of essential oils, which destroys bacterial cell membranes and allows nutrients to flow out of cells, so as to achieve the purpose of sterilization.

#### 2.2.1 Chitosan

There is a large amount of chitin in the crustaceans of marine arthropods and insects such as shrimp and crabs. Also, it could be found in the cell membranes of fungi and algae, the shells and bones of molluscs and the cell walls of higher plants. Chitin produces positively charged cationic groups in the reaction, which is the only positively charged edible food fiber in nature. Under the combined action of chitosan aminotransferase, deacetylase (existing in plants and intestinal bacteria), lysozyme (existing in the body) and lecithin (existing in milk and eggs), chitin can be decomposed into oligosaccharides, thus being absorbed by the human body.

Chitosan is the product of natural polysaccharide chitin that removes part of the acetyl group. It has a variety of physiological functions such as biocompatibility, non-toxic, bacteriostasis, anti-cancer, lipid reduction, and immune enhancement. It is widely used in food additives, textile, agriculture, environmental protection, beauty care, cosmetics, antibacterial agents, medical fibers, medical dressings, artificial tissue materials, drug slow-release materials, gene transduction carriers, biomedical fields, medical absorbable materials, tissue engineering carrier materials, medical and drug development and many other fields and other daily chemical industry. In the field of food packaging, they act as natural food antibacterial agents that inhibit the biological activity of molds, yeasts, Gram-positive and Gram-negative bacteria.^68^ However, the poor solubility of chitosan at neutral and alkaline pH limits its application in the field of food preservation.

Chitosan packaging film can protect food through various mechanisms. Fig. 3 shows the different antimicrobial mechanisms of chitosan. It can limit the respiratory activity of microorganisms by blocking oxygen and may form a physical barrier that prevents them from accessing nutrients (Mechanisms I and II). Chitosan chains can chelate various nutrients (Mechanism III), and the electrostatic interaction between chitosan cationic groups and anionic bacterial cell membranes can damage bacterial cell membranes, resulting in cell membrane rupture, intracellular fluid outflow, and bacterial death (Mechanism IV). Chitosan may also act on cell membranes, causing damage to cell membrane's function and structure, which will lead to leakage of intracellular components (Mechanism V). In addition, it can chelate nutrients in cell plasma or trace metal ions (Fe^3+^) necessary for cell metabolism, thereby affecting cell metabolic processes (Mechanism VI). It may also promote the synthesis of chitinase (chitinase can degrade fungal cell walls), thus affecting gene expression (Mechanism VII) or low molecular weight chitosan may enter the nucleus and bind to DNA, thereby inhibiting the microbial proliferation process (Mechanism VIII).^68^

**Fig. 3:**
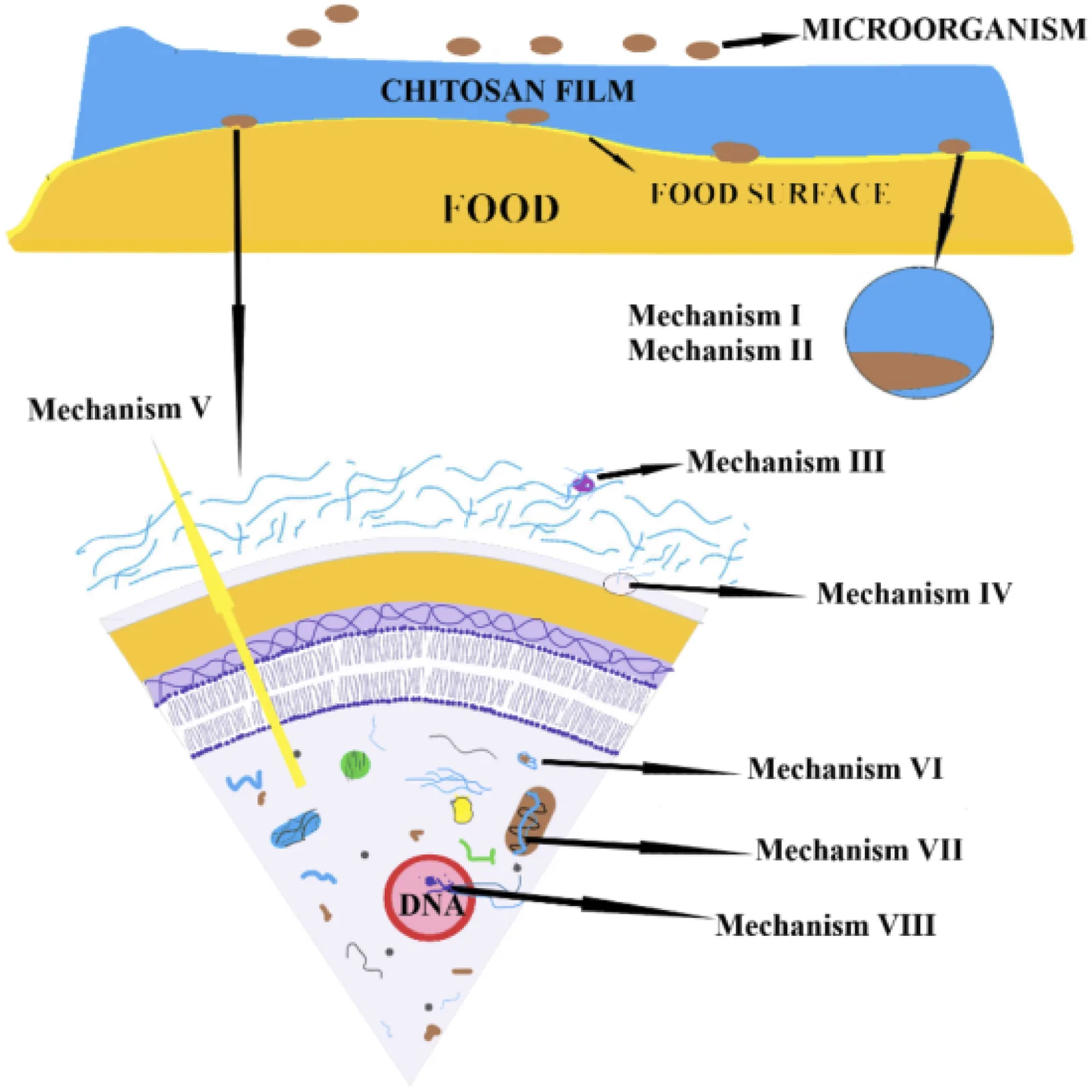
The antimicrobial mechanism of chitosan.^69^ Reproduced from ref. 69, Motelica *et al.*, Biodegradable Antimicrobial Food Packaging: Trends and Perspectives, *Foods*, 2020, **9**(10), 1438, https://doi.org/10.3390/foods9101438, licensed under CC BY 4.0.

There are many types of films based on chitosan. At present, the common forms include pure chitosan film, chitosan/biopolymer film, chitosan/synthetic polymer film, and chitosan derivative film. Vacuum packaging is often combined with pure chitosan film for food preservation, such as swordfish, cod, perch and rainbow trout.^70,71^ In addition, some plasticizers such as glycerin and sorbitol, or emulsifiers such as Tween 80, Tween 20 and Span 20, are added appropriately during the preparation of chitosan film to improve the strength, toughness and other mechanical properties.^72^ At the same time, chitosan can also be combined with polysaccharides, proteins and other natural extracts that are biodegradable, non-toxic and biocompatible to prepare films, which are also proved to have antibacterial effects when used in food packaging. For example, the composite film made by combining chitosan and gelatin exhibits excellent antioxidant and water vapor barrier properties, which reduces the adsorption of bacteria on food packaging.^68^ The films can also be prepared by combining chitosan with alginate, a natural anionic polysaccharide with favorable properties such as low toxicity and chemical versatility. The composite film was developed by electrostatic deposition technology based on the opposite charges of chitosan and alginate themselves, and the research results showed that the film had excellent gas exchange performance and water vapor permeation performance, showing great potential in the field of food packaging.^73,74^ In addition, the combination of chitosan film and plant extract can significantly improve the film properties through the synergistic effect of the two, such as antimicrobial activity (naringin extract^75^), antioxidant activity (eugenol,^76^ mackey berry extract^77^), barrier properties (thyme extract^78^), mechanical properties (tannin^79^), thermal stability (apple polyphenols^80^), and color properties (carvacrol^81^). Priyadarshi, R. *et al.* combined chitosan with citric acid and used glycerin as a plasticizer to enhance the stability and antioxidant properties of the film while also making it flexible. The prepared film has a transparent appearance, increased flexibility and decreased water vapor permeability, which can effectively extend the shelf life of green chill peppers.^82^ The chitosan/cassava starch film prepared by shiam, MAH *et al.* using subcritical water technology has reduced water vapor permeability and extended the shelf life of ham by 18 days, showing good antibacterial effects.^83^

The combination of chitosan and essential oils is currently one of the research hotspots. Although this type of packaging material has good biological properties, it should be noted that the type of essential oil used needs to match the type of food being protected. For example, thyme, ginger or rosemary essential oils are favored by consumers in meat products, but their effect will be greatly reduced when they are paired with fruit; cinnamon and vanilla work well with pastries, but are less satisfying with meat; the essential oil of oranges or limes can be used in fruit or seafood packaging.

These literatures described the types of essential oils applicable to different foods, and also showed that the combination of chitosan with other biopolymers or essential oils can be used as one of the strategies to effectively improve food quality and extend the shelf life of commodities, which is currently a major research focus for researchers to apply to food antibacterial materials at present.

#### 2.2.2 Lysozyme

Lysozyme is regarded as GRAS, can be directly contained in the food substrate and has a significant inhibitory effect on some microorganisms. Lysozyme shows good stability at different temperature ranges (4–95 °C) and different pH ranges (2–10), and it has a strong effect on Gram-positive bacteria, such as *Listeria monocytogenes*, *Bacillus lipophilus thermophilus*, *Micrococcus* and *Clostridium butyricum*. However, the antibacterial efficacy against Gram-negative bacteria is far less than that of Gram-positive bacteria. This is because the antimicrobial activity of lysozymes is attributed to the fact that they can accelerate the hydrolysis of the bond between *n*-acetyl-muramic acid and *n*-acetyl-d-glucosamine, thereby destroying the peptidoglycan layer of the bacterial cell wall. However, Gram-negative bacteria have lipopolysaccharide layer, which inhibits lysozyme from entering the site of action.^84^ In this regard, researchers have proposed various methods to improve the antibacterial efficacy of lysozyme against Gram-negative bacteria, including denaturation of the enzyme, modification of lysozyme by connecting with other compounds, and the use of membrane permeating agents for lysozyme.^85^ These techniques involve covalent linking of saturated fatty acids and lysine residues, denaturation due to temperature effects, reduction of disulfide bonds, glycosylation and chelating compounds, *etc*.^86^ As show in Fig. 4, four chemical modification approaches to enhance lysozyme antibacterial activity against Gram-negative bacteria. Lysozyme–chitosan films can also improve the freshness of eggs during storage, improve eggshell strength, and maintain the internal quality.^87^

**Fig. 4:**
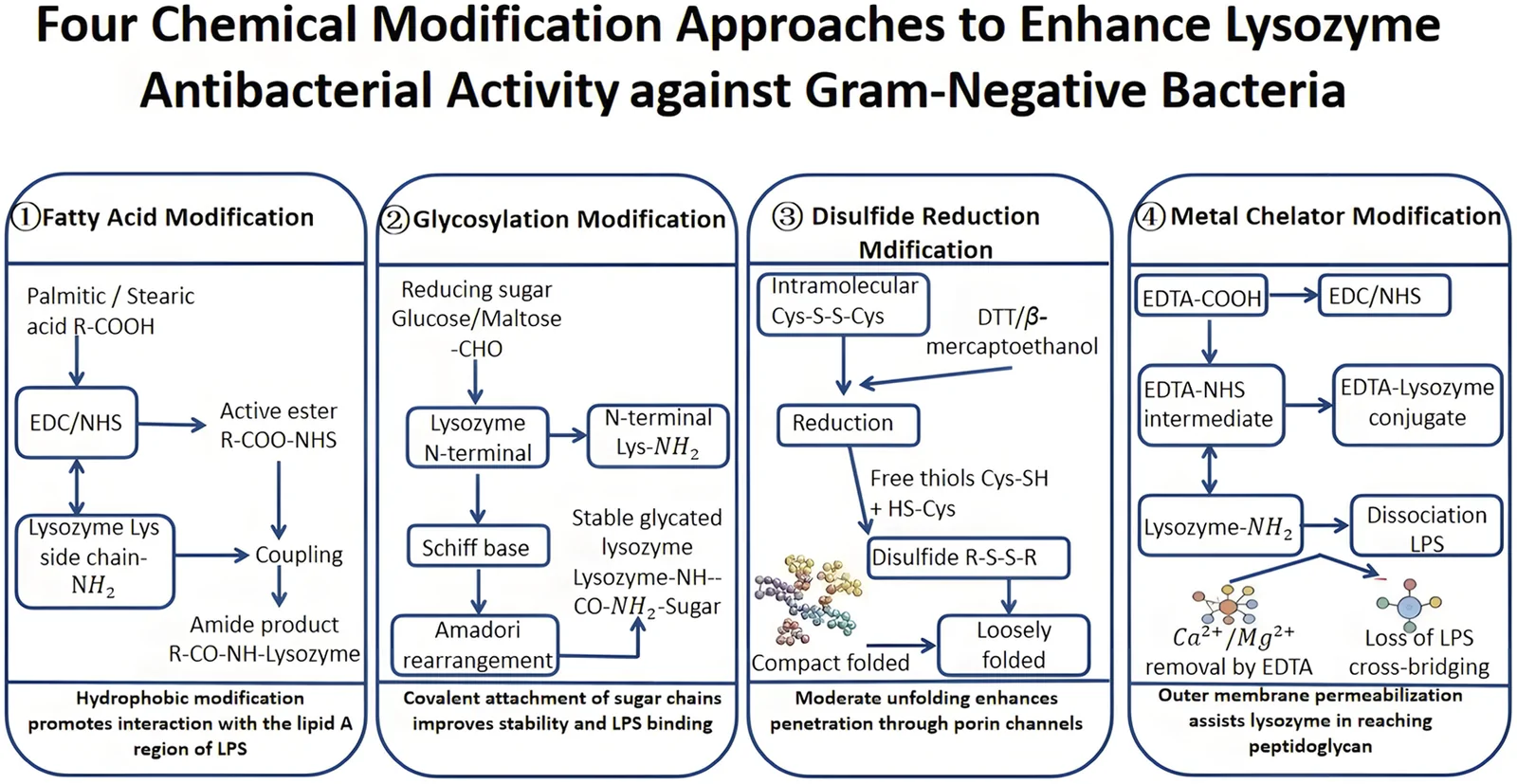
Four chemical modification approaches to enhance lysozyme antibacterial activity against Gram-negative bacteria. (1) Fatty acid modification; (2) glycosylation modification; (3) disulfide reduction modification; and (4) metal chelator modification.

#### 2.2.3 Lactoferrin

The researchers found that lactoferrin in milk has an antibacterial effect and can be used to inhibit the growth of spoilage and pathogenic microorganisms in food. At the same time, researchers also found that this globular glycoprotein component also has antioxidant, anti-cancer, anti-obesity and other properties.^88^ In addition, lactoferrin exists in the secretions of the digestive system, reproductive system and respiratory system of humans and animals, which shows that lactoferrin has a very good compatibility with the human body, and will not have side effects on human health while exerting its antibacterial effect. Lactoferrin has significant antibacterial action against both Gram-positive and Gram-negative bacteria, viruses and fungi. Its antibacterial mechanism is to reduce the absorption of nutrients by microorganisms through iron chelation, thus leading to the destruction of bacterial cell membranes and inhibiting microbial activity.^89^ Lactoferrin, a globular glycoprotein of the transferrin family, consists of two homologous globular lobes (N- and C-lobe), each containing a high-affinity Fe^3+^ binding site. Each site sequesters one Fe^3+^ ion synergistically with a bicarbonate co-ligand: four primary coordination bonds are donated by conserved tyrosine, aspartic acid and histidine residues, and two additional oxygen ligands from bicarbonate form a stable six-coordinate octahedral geometry. With a binding constant on the order of 10^20^ M^−1^ and near-irreversible binding under physiological pH, lactoferrin effectively scavenges free ferric iron from the microbial microenvironment.

Iron acts as an essential cofactor for a range of microbial iron-dependent enzymes (*e.g.* cytochrome oxidase, catalase, ribonucleotide reductase) that govern core processes including respiratory electron transport, DNA biosynthesis and cellular antioxidant defence. Lactoferrin-mediated iron sequestration induces an iron-starvation state in the microbial habitat, impairing the biosynthesis of iron-containing enzyme cofactors. This in turn blocks respiratory electron transport, suppresses cellular energy production, inhibits nucleic acid synthesis and cell proliferation, and disrupts iron-dependent antioxidant systems. The resulting elevated intracellular oxidative stress further compromises bacterial membrane integrity, ultimately exerting antimicrobial effects.

It has been reported that encapsulation of curcumin in lactoferrin/pectin matrix can increase the water solubility of hydrophobic curcumin and control the release of curcumin.^90^ When 1 mg mL^−1^ lactoferrin was incubated at 37 °C for 8 h, the count of *Listeria monocytogenes* was reduced by up to 4 Log.^91^ In addition, lactoferrin can also be coated with lysozyme in the membrane matrix, which can effectively inhibit bacterial activity in salmon fillets.^92^

#### 2.2.4 Lactoperoxidase

Lactoperoxidase (LPS), like lactoferrin, is abundant in milk and in body fluids such as tears and saliva. LPS exhibits good inhibitory properties against many bacteria such as salmonella, pseudomonas and *Escherichia coli*. Lactoperoxidase can catalyze thiocyanic acid ion (SCN–) to produce hypo thiocyanic acid (OSCN–) and hypo thiocyanic acid (HOSCN).^93,94^ These two have antibacterial activity due to oxidation of the sulfhydryl (SH) groups of proteins.^95^ Due to its wide range of sources, lactoperoxidase is relatively cheap and easy to obtain. Its antibacterial properties have great application prospects in the field of food packaging, but its application in meat products is still being continuously explored.

### 2.3 Antibacterial agents of microbial origin

Some microorganisms and their derivatives also have the ability to inhibit the reproduction and growth of spoilage microorganisms and bacteria in food. At the same time, they will participate in the competition of nutrients and *Lebensraum*, which will extend the shelf life of food and ensure that the quality and flavor of food will not be changed. The application of these antagonistic microorganisms and their derivatives in the food field is called biological preservation.^96^ Lactic acid bacteria is one of the representatives of these antagonistic microorganisms. As an antagonistic microbe, it can compete with spoilage microorganisms in food and produce nutrients and metabolic compounds with antimicrobial properties.^97^ The US Food and Drug Administration (FDA) has classified lactic acid bacteria as GRAS, and the European Food Safety Authority (EFSA) has also classified lactic acid bacteria as Qualified Presumption of Safety (QPS).^98^ At present, lactic acid bacteria are mostly used in fresh fruits and vegetables,^99,100^ but they are easily affected by many factors, such as pH, food types, temperature, *etc.* For example, at low pH value, the growth of lactic acid bacteria will be inhibited.^101^

#### 2.3.1 Bacteriocins

Bacteriocins have good antibacterial effects on microorganisms in less processed products such as fruits and vegetables, especially Gram-positive bacteria; and the antibacterial agent produced by them has the advantage resistance to high temperature and acid environments. From a structural chemistry perspective, bacteriocins are a class of amphiphilic cationic peptides. Their primary sequences are enriched in positively charged amino acid residues (*e.g.*, lysine and arginine) that are spatially segregated from hydrophobic amino acid domains, conferring distinct molecular amphipathicity. The net positive charge enables strong electrostatic attraction to negatively charged components on the bacterial cell surface: for Gram-positive bacteria, the anionic phosphate groups of wall teichoic acids and lipoteichoic acids serve as the main binding sites; for Gram-negative bacteria, the negatively charged carboxylate and phosphate groups of lipopolysaccharide (LPS) in the outer membrane mediate the initial electrostatic docking. This charge-driven adhesion concentrates bacteriocin molecules on the membrane surface and facilitates the insertion of their hydrophobic domains into the lipid bilayer. Following insertion, the hydrophobic peptide segments assemble and oligomerize within the membrane to form transmembrane pores. Pore formation disrupts membrane integrity, dissipates transmembrane ion gradients, causes leakage of intracellular metabolites and ions, and ultimately leads to bacterial cell death.^102^ At present, researchers tend to combine them with other antibacterial measures to achieve better antibacterial effects. For example, bacteriocins can be assembled with existing food packaging materials, such as directly incorporated into the polymer or coated on the polymer surface, so as to prevent food deterioration, extend the shelf life of food, and ensure food quality.

#### 2.3.2 Nisin

Lactostreptin, a bacteriocin isolated from *Lactococcus lactis* strains, is an amphiphilic cationic peptide. Approved by FDA it is considered as GRAS antibacterial agent, so it will not pose any harm to human body when used for food preservation.^103^ It is not only resistant to high temperatures, but also has significant antibacterial properties against broad spectrum Gram-positive bacteria, such as *Staphylococcus*, *Bacillus cereus*, *Lactobacillus*, monocyte proliferating *Listeria monocytogenes*, *Clostridium botulinum*, *Micrococcus* and *Pediococcus*. However, its inhibitory ability against Gram-negative bacteria is not very obvious.^104,105^ The antibacterial mechanism of nisin can be attributed to its ability to bind to the cell wall of specific bacteria and subsequently form pores in the cell membrane, resulting in the outflow of intracellular nutrients, thus achieving the purpose of inhibiting bacterial activity.^106^ According to literature reports, due to the uniform distribution of nisin in liquid systems, its activity is more effective in liquid foods than in solid foods. At present, nisin and other bacteriocins have shown significant inhibitory effects on the activities of *Clostridium botulinum* and *Listeria monocytogenes* in cheese, and the combination of nisin and eumycin has certain inhibitory effects on yeast and mold in edible olive oil.^7^

#### 2.3.3 Bacteriophage

Bacteriophages are also a type of antibacterial agent. It can be fully applied to the disinfection of meat, fruits, vegetables and other products, so as to achieve the prevention and suppression of microbial colonies and extend the shelf life of food. At the same time, bacteriophage biological control methods can also be used to extend the shelf life of food, which can serve as symbiotic organisms of fermentation microorganisms as biological preservatives during the fermentation process.^17^ Sezer *et al.* produced four films by combining a mixture of three bacteriophages with four different biopolymers (WPC, carboxymethyl cellulose, chitosan, and sodium alginate) and found significant inhibition on the activity of *Salmonella enteritidis* and *Salmonella typhimurium* in strawberries stored at 4 °C for 5 days. Among them, strawberries coated with WPC had the most obvious inhibitory effect, followed by strawberries coated with chitosan, sodium alginate and carboxymethyl cellulose.^107^ Bacteriophages can inhibit the growth of *Listeria monocytogenes* in Turkey^108^, *Escherichia coli* in meat^109^ and *Staphylococcus aureus* in cheese.^110^ In addition, Rindhe applied edible films based on methyl cellulose and encapsulated bacteriophages to raw fish slices, and proved that it had obvious inhibitory effect on *Vibrio parahaemolyticus* in raw fish slices stored at 4 °C for 14 days.^111^

#### 2.3.4 Reuterin

Reuterin is produced by a strain called *Lactobacillus reuteri* in the presence of glycerol, and has significant antibacterial properties against a variety of pathogenic and putrefying microorganisms, such as *Listeria monocytogenes*, *Staphylococcus aureus*, *Salmonella choleraesuis*, *Escherichia coli*, and *Aeromonas hydrophila*.^4^ According to literature reports, there are two main antibacterial mechanisms of it. One is that the aldehyde group contained in it can react with the sulfhydryl group of nutrients required for bacterial growth. Second, it can affect the synthesis of DNA to achieve the purpose of sterilization.^112^ The film prepared by combining *Lactobacillus royi* with glycerin and sodium alginate was used to preserve sea bass fillets, it was found that it showed good antibacterial activity and resulted in a significant decrease in the activity of major spoilage microorganisms.^113^ At present, there is also literature showing that reuterin has a significant antibacterial effect on *Salmonella enterica* in broilers.^114^ In addition, reuterin was found to have an effective inhibitory effect on the activity and quantity of *Pseudomonas*, *Listeria monocytogenes* and *Escherichia coli*.^112^ Therefore, reuterin also holds great promise in the field of food antibacterial.

#### 2.3.5 Pediocin

Pediocin is also produced by a strain of *Lactobacillaceae*, which has a random coil model in aqueous solutions, but in some non-aqueous solutions, it has a partially helical structure. The activity of pediocin is easily affected by temperature, pH, salinity within the food, enzyme activity (such as proteolysis and storage period).^17^ The antibacterial effect of pediocin lies in the fact that it can form voids in the bacterial cytoplasm, which imbalances the inherent pH of the bacterial cell, thus inactivating the proteins responsible for energy production in the cell.^115,116^ Literature has shown that the nano-composite food packaging materials prepared by combining methylcellulose with pediocin and nano-Zno have significant antibacterial effects on *Listeria monocytogenes* and *Staphylococcus aureus*, and the mechanical properties of the prepared films have also been improved.^117^ In addition, pediocin's antibacterial properties against fermented sausages, as well as some vegetables and dairy products, are also very impressive.^118–121^ Pediocin has great potential in the antibacterial packaging field for food, and it has a great prospect for food preservation and shelf life extension.

## 3 Antibacterial packaging type

Antibacterial food packaging is to introduce antibacterial agents which is harmless to the human body into the packaging material, inhibit the growth of microorganisms, extend the shelf life of food, thereby the packaging material has antibacterial function. Antibacterial packaging can be divided into the following types: (1) add antibacterial bags to the packaging material; (2) direct coating of antibacterial agents; (3) encapsulation antibacterial packaging; (4) absorbent antibacterial packaging.

### 3.1 Add antibacterial bags to the packaging material

Adding antibacterial small bags to packaging materials is suitable for some volatile antibacterial agents. While fully utilizing the antibacterial properties of antibacterial agents, it can also avoid direct contact between antibacterial agents and food, ensuring the quality, safety, and flavor of food are not affected. In this technical route, volatile natural antimicrobials (predominantly essential oils) are first complexed with host carrier materials such as β-cyclodextrin to form chemical inclusion complexes, and then the carrier-active agent composite is packed into a permeable non-woven or cellulose-fiber pouch, which is subsequently sealed and placed inside food packages. Antibacterial function is realized *via* sustained release of volatile antibacterial compounds into the package headspace to inhibit airborne and surface-attached bacterial growth.^122^ Release kinetics of volatile bioactive compounds from host–guest inclusion complexes within the package headspace is a core performance indicator for this antibacterial-sachet system. β-Cyclodextrin, as a cyclic oligosaccharide host molecule, possesses a hydrophobic inner cavity and hydrophilic outer surface. Volatile essential-oil guest molecules can enter the cavity through host–guest intermolecular interactions including hydrophobic effect, van der Waals forces and hydrogen-bonding interactions to form solid-state inclusion complexes. This chemical inclusion effect greatly stabilizes volatile essential oils: it reduces the high volatility of raw essential oil molecules, restrains their rapid spontaneous sublimation, protects active terpenoid and phenolic components against thermal degradation, photo-oxidation and environmental evaporation loss during storage. Without such host–guest encapsulation, neat essential oils would undergo fast, uncontrolled sublimation, leading to burst release at the early storage stage, rapid depletion of active components, and loss of long-term antibacterial efficacy.

The host–guest binding strength determines the sublimation and release rate of essential-oil components. Weak host–guest affinity facilitates faster dissociation of the inclusion complex, accelerating volatile-compound sublimation and headspace concentration build-up; stronger binding interaction slows complex dissociation, suppresses sublimation rate, and extends the release duration of volatile antimicrobials. Accordingly, adjusting the molar ratio of β-cyclodextrin to essential-oil guest, reaction preparation conditions, and modifying cyclodextrin substituents can purposefully tune release kinetics, maintaining an effective yet non-excessive volatile-antimicrobial concentration in the package headspace over the whole shelf-life period. Excessively high headspace volatile concentration would bring undesirable off-flavor to food, while insufficient release would fail to achieve satisfactory bacteriostatic effects.

For example, Clara *et al.* used β-cyclodextrin to encapsulate basil essential oil and *Dioscorea* essential oil respectively, and then put 0.5 g of the inclusion compound into a sterilization bag composed of cellulose fiber, and conducted *in vitro* bacteriostatic test on the prepared antibacterial bag. They found that it had obvious bacteriostatic effect on *Bacillus* and foodborne *Listeria monocytogenes*.^123^ Narumol *et al.* placed (lavender) MA essential oil in small bags of three different materials to observe its inhibition effect on mold in brown rice and its controlled release effect.^124^ Yunilawati *et al.* prepared β-cyclodextrin/lemongrass essential oil inclusion compound by co-precipitation method. 1 g inclusion compound was filled into the non-woven pouch, and then the pouch was placed in the package of fresh shrimp and stored at room temperature for 30 h to monitor the degree of shrimp spoilage. The results showed that the inclusion compound exhibited significant antibacterial activity against *Escherichia coli* and *Staphylococcus aureus*, and the inclusion compound showed sustainable release of active compounds.^125^ At present, this antibacterial method is simple, effective and low cost, and has been widely used in commercial aspects.

### 3.2 Direct coating of antibacterial agents

Directly coating antibacterial agents on films is currently also a very common type of antibacterial packaging. By applying antibacterial agents directly, an edible coating is formed on the surface of food to prevent the growth of spoilage microorganisms. The components of edible coatings are different, but the functions are mostly similar, and they can achieve the purpose of blocking water vapor, oxygen and inhibiting microorganisms. Most food coating components can be divided into: lipids, hydrocolloid, composite materials. Lipids include acyl, glycerol and fatty acids. Hydrocolloids include polysaccharides and proteins, such as alginate, starch, chitosan, cellulose derivatives and ager. Composite materials usually include both of the above two types.^126^ For example, Ghosh applied the functionalized nanocellulose–curcumin–chitosan food coating developed on kiwifruit, and the results showed that nanocellulose and curcumin could provide synergistic effects in maintaining the quality of kiwifruit, effectively reducing the mass loss, hardness loss, respiratory rate and microbial population of kiwifruit during storage.^127^ Hu *et al.* developed edible coatings on strawberries and the developed coatings consisting of *trans*-resveratrol encapsulated nanoparticles embedded in a chitosan matrix. The results showed that the edible coating could significantly prolong the storage period of strawberries, effectively prevent the loss of nutrients of strawberries and maintain the quality of strawberries.^128^ Wang *et al.* prepared chitosan based food coating by adding γ-polyglutamic acid and curcumin, and the results showed that compared with pure curcumin-based active coating, chitosan/γ-polyglutamic acid/curcumic edible coating significantly extended the shelf life of fresh beef and effectively ensured the freshness of beef.^129^

Notably, the stability and functional performance of this ternary coating originate from non-covalent intermolecular interactions among chitosan, γ-polyglutamic acid and curcumin. Under the slightly acidic conditions typically adopted for edible coating formulation, the protonated amino groups (–NH_3_^+^) of chitosan undergo electrostatic complexation with the deprotonated carboxylate groups (–COO^−^) of γ-polyglutamic acid, affording a compact polyelectrolyte complex network that constitutes the structural skeleton of the coating matrix. Concurrently, the hydroxyl and amino groups along the biopolymer chains establish extensive hydrogen bonding with the phenolic hydroxyl moieties of curcumin. These synergistic interactions firmly anchor the hydrophobic curcumin within the hydrophilic polysaccharide network, suppressing molecular self-aggregation, improving dispersion uniformity, and mitigating photodegradation and aqueous leaching of the active compound. Consequently, the physical integrity and long-term bioactivity of the edible coating are preserved during storage. In the absence of such electrostatic and hydrogen-bonding cross-links, curcumin would be prone to rapid phase separation and leaching, resulting in deteriorated coating performance and a shortened effective antibacterial period.

### 3.3 Encapsulation antibacterial packaging

Encapsulation antibacterial packaging is often associated with nanotechnology. Embedding different active ingredients into other polymer substrates and releasing them under specific conditions, such packaging technology can realize the sustain release of antibacterial agents, better utilize the antibacterial properties without contact with food, avoid the waste of antibacterial agents, and ensure the flavor and safety of food.^130^ Chemical cross-linking represents a critical fabrication step in encapsulated antibacterial packaging, as it governs encapsulation efficiency, barrier performance and the release behaviour of active compounds. Two food-grade strategies are predominantly employed: ionotropic gelation of alginate with Ca^2+^ and transglutaminase-mediated enzymatic cross-linking of gelatin. During alginate gelation, Ca^2+^ bridges adjacent guluronate blocks to form “egg-box” junction zones, providing a mild, solvent-free encapsulation matrix. Moderate Ca^2+^ levels afford a compact network that restricts payload leakage and enhances oxygen and moisture barrier properties, whereas excess Ca^2+^induces over-cross-linking, matrix shrinkage and micro-crack formation, thereby reducing encapsulation efficiency and barrier performance. In enzymatic gelation, transglutaminase catalyses the formation of ε-(γ-glutamyl)-lysine isopeptide bonds between glutamine and lysine residues of gelatin, generating covalent cross-links with superior thermal and water resistance compared with ionic gelation. Appropriate enzyme dosage and incubation conditions yield uniform networks that efficiently entrap active payloads and lower water-vapour permeability, while insufficient or excessive enzyme results in loose matrices or excessively rigid, defective structures, respectively. Notably, cross-linking conditions (temperature, pH, time) should be carefully optimised to preserve thermo-labile antibacterial agents such as bacteriocins and essential-oil components.

In recent years, packaging technology is more and more widely used in the field of food antibacterial packaging. For example, silver nanoparticles are encapsulated in silica matrix. Compared with non-encapsulation, the encapsulation of silver nanoparticles avoids agglomeration and self-oxidation phenomenon, improves the antibacterial property of silver nanoparticles, and nano-fillers can also improve some properties of composite films.^131^ In addition, Marwa *et al.* found that the edible packaging coating of thyme essential oil encapsulated by chitosan could significantly improve the shelf life of cheese.^132^ Literature has shown that encapsulation of essential oils into sodium alginate and gelatin can effectively avoid the loss of active ingredients in essential oils (80% of their original content).^133^ The experiments by Viacava *et al.* proved that the addition of microcapsules of thyme did not have any negative effect on the sensory properties of lettuce, and the scent of the essential oil did not come out.^134^ Norcino *et al.* added copaiba oil nanoemulsion in the process of pectin film preparation, and then formed the film through continuous casting and drying to observe the significant antibacterial activity of the film against *Staphylococcus aureus* and *Escherichia coli*. Moreover the essential oil significantly improved the mechanical properties of the film.^135^ The slow-release properties of clove essential oil were observed when nanoemulsions containing clove essential oil were combined with gelatin films.^136^ Zhou *et al.* used high shear homogenization and ultrasonic treatment to simultaneously encapsulate camellia essential oil and produce glucomannan/carrageenan-based edible films.^137^ The experiment showed that encapsulation of essential oil in the film did not affect the odor of chicken, further confirmed the advantages of encapsulated antibacterial packaging. Frine *et al.* made microcapsules containing 2.5% and 5% thymol or carvacrol in β-cyclodextrin, which were then mixed with PLA and injection molded to prepare the packaging box. The package significantly extend the shelf life of blackberries and raspberries to 21 days. Moreover, PLA packaging containing 5% β-cyclodextrin-thymol has a higher inhibition rate against yeast and mold.^138^ The packaging film prepared by combining oregano essential oil/β-cyclodextrin with PLA/PCL can significantly extend the shelf life of blackberry.^139^ These literature has shown that polysaccharides have good barrier properties to O_2_ and CO_2_, so they can delay the ripening of some vegetables and fruits by inhibiting their respiratory activities.^126,140,141^

Active packaging, edible coating, intelligent packaging, biodegradable packaging are all new technologies for packaging materials at present.^142^ Antibacterial compounds can be absorbed during the preparation process of the package and exert their own antibacterial properties at appropriate times and under appropriate conditions.^143^

### 3.4 Absorbent antibacterial packaging

Some fresh meat products will produce some exudates such as moisture in the storage and transportation process, which may have adverse effects on product quality, flavor and so on. Therefore, absorption pads and other structures can be used to solve this problem. Place the absorption pad at the bottom of the tray or container to absorb the moisture that may seep out of the food during storage. In order to ensure that the absorbent pad does not affect the quality and flavor of the product, researchers have made the following requirements for the structure of the absorbent pad:^122^

(1) The absorbent pad should be placed at the bottom of the product, without affecting the appearance of the product, and the absorbent pad must have a structure that does not leak liquid;

(2) When taking out the absorbent pad, it should be ensured that the liquid in the absorbent pad will not flow out to avoid affecting the quality and safety of food;

(3) The cost of absorption pads needs to be controlled, which also reduces the economic burden on consumers;

(4) The addition of absorption pads should extend the shelf life of food and ensure its flavor.

The core water-absorbing components within absorbent layers mainly comprise super-absorbent polymers, cellulose fibres and modified starch. These biopolymer-based materials realise rapid liquid uptake and liquid retention *via* capillary action together with polymer–water intermolecular interactions. Porous physical frameworks constructed by intertwined cellulose fibres and modified-starch granules generate abundant capillary channels, where capillary force drives rapid wicking of food exudates into the interior of absorbent media. Meanwhile, hydrophilic polar groups (–OH, –COO^−^) distributed along polymer chains form hydrogen-bond interactions with water molecules. Super-absorbent polymers further swell upon liquid contact, forming hydrated cross-linked networks that physically trap water inside matrix voids and prevent liquid re-leakage under external compression. The synergy of capillary wicking effect and hydrophilic polymer–water interactions enables fast absorption of meat exudates and stable liquid locking, avoiding secondary contact between free liquid and food surfaces.

Usually, absorbent pads can be divided into three layers, namely the upper, middle and lower layers, similar to the mechanism of capillary action. The lower layer is usually composed of an impermeable thermoplastic material, or covered with a cellulose-like material.^144^ This cellulosic material can come into direct contact with food without posing any threat to food quality and safety. In addition, researchers create specific piercings at a distance between the upper and lower layers, so that they can properly absorb the liquid and water in the food without compromising food flavor and quality safety.^145^ For example, Yavari Maroufi *et al.* developed a chitosan-modified aldehyde guar gum (DAGG) hydrogel, which showed that the prepared hydrogel had a porous structure. The hydrogel containing 5% pomegranate peel extract (PPE) had good antioxidant activity (81.13%) and antibacterial activity, and it has the potential to be used as an antibacterial pad.^146^ Chen *et al.* prepared a composite double-layer film based on corn starch/polylactic acid (PLA). The film has a hydrophobic outer layer and an absorbent inner layer. The incorporation of eucalyptus essential oil microcapsules into the film greatly improved the antioxidant and antibacterial properties of the double-layer film. It effectively inhibited the respiration rate of the mushroom, slowed down the consumption of organic matter, and greatly extended the shelf life of the mushroom.^147^

Although natural antibacterial agents already exist in many different antibacterial packaging, there are still some challenges and limitations, such as the lack of compatibility between polymer substrates and antibacterial agents, the migration of antibacterial agents into food, and the safety of antibacterial agents themselves. These require researchers to conduct more in-depth research and development on natural antibacterial agents and antibacterial packaging types.

## 4 Conclusion

Consumers' expectations for food quality are gradually increasing, with the hope that food processing will be fewer, cleaner, and with lower additive content. This requires researchers to develop multiple types of food packaging materials. In the development process, it is also necessary to consider various other factors such as material source, bio-compatibility, lifespan, food preservation mechanism, recycling strategy and environmental pressure. For example, in antibacterial active packaging, we need to consider the source of polymer matrix materials and the source of antibacterial agents. Moreover, we must consider the safety issues, regulatory regulations, sensory properties and consumer acceptance of packaging materials and antibacterial agents. At the same time, the extraction of antibacterial agents also needs to pay attention to factors such as temperature and method to avoid the failure of antibacterial agents.

The emergence of natural antibacterial agents has brought greater and broader prospects for food packaging. They come from nature, do not put any pressure on the environment, with almost zero side effects on the human body. At present, although many natural antibacterial agents have emerged, there is still a lot of work to be done in a series of procedures such as the extraction, processing, optimization, industrial-scale application, and the suitability of different types of food with natural antibacterial agents. While biopolymer-based antibacterial packaging incorporated with natural antimicrobials exhibits promising lab-scale performance, substantial translational gaps persist between laboratory-level demonstrations and practical industrial deployment. A structured future research roadmap is therefore proposed targeting three core bottlenecks: manufacturability for industrial-scale upscaling, techno-economic evaluation of natural antimicrobial production, and harmonised safety-regulatory frameworks for food-contact active materials. Current studies are predominantly confined to laboratory-batch fabrication. Continuous manufacturing workflows including roll-to-roll film casting, large-volume ionotropic gelation and industrial enzymatic cross-linking should be further optimised to adapt to existing food-packaging production infrastructure. Special attention needs to be devoted to batch-to-batch performance deviation induced by intrinsic heterogeneity of bio-derived raw feedstocks. Systematic characterisation of finished-product stability under realistic logistics conditions (fluctuating temperature, humidity and light exposure), alongside pilot-plant trials, is essential to generate industrially valid datasets prior to full commercialisation. High feedstock and downstream-processing costs remain major obstacles restricting real-world implementation of plant-, animal- and microbial-sourced antimicrobials. Beyond laboratory-only performance evaluation, techno-economic assessment (TEA) ought to be integrated into the whole material development workflow. Valorisation of agro-industrial by-products such as fruit peels and fermentation residues offers an attractive pathway to cut raw-material expenditure. Further refinement of low-energy green extraction protocols is required to balance extraction yield, antimicrobial bioactivity and manufacturing expenditure, enabling efficient screening of formulations that satisfy both technical functionality and commercial economic constraints.

At the same time, the trade-off between antibacterial potency and food sensory attributes deserves sustained attention, as off-flavours originating from essential-oil-related constituents represent a notable barrier to consumer acceptance. Ultimately, multi-criteria decision analysis integrating functional performance, techno-economic feasibility, toxicological safety, sensory acceptability and end-of-life biodegradability will facilitate the practical translation of next-generation biopolymer-based antibacterial food packaging.

## Author contributions

E. D.: conceptualization, methodology, investigation, data curation, visualization, writing – original draft. Y. Y.: investigation, data curation, visualization, writing – review & editing. Y. X.: conceptualization, methodology, supervision, project administration, funding acquisition, writing – review & editing. Y. W.: conceptualization, supervision, resources, funding acquisition, writing – review & editing. All authors have read and agreed to the published version of the manuscript.

## Conflicts of interest

There are no conflicts to declare.

## Data Availability

No primary research results, software or code have been included and no new data were generated or analysed as part of this review.
